# Gut microbiome in type 2 diabetes: insights from metagenomics, multi-omics, and diet–microbe interactions

**DOI:** 10.1080/19490976.2026.2644682

**Published:** 2026-03-17

**Authors:** Yu Zhang, Dong D. Wang

**Affiliations:** a Department of Nutrition, Harvard T.H. Chan School of Public Health, Boston, USA; b Broad Institute of MIT and Harvard, Cambridge, USA; c Channing Division of Network Medicine, Department of Medicine, Brigham and Women's Hospital and Harvard Medical School, Boston, USA

**Keywords:** Type 2 diabetes, microbiome, diet, metabolomics, metagenomics, metatranscriptomics, microbial strains

## Abstract

Type 2 diabetes (T2D) is a heterogeneous metabolic disorder in which environmental exposures interact with host biology to drive insulin resistance and progressive *β*-cell dysfunction. This review synthesizes recent advances showing how the gut microbiome mediates these processes across multiple levels of resolution. First, large-scale shotgun metagenomic studies consistently identify a reproducible T2D-associated signature characterized by depletion of short-chain fatty acid–producing taxa and enrichment of opportunistic, pro-inflammatory microorganisms, while highlighting the importance of controlling for major confounders such as adiposity and glucose-lowering medications. Second, functional profiling and metabolomics link microbial community shifts to coordinated pathway changes—including reduced short-chain fatty acid and secondary bile acid production and increased endotoxin- and branched-chain amino acid–related metabolism—that influence gut barrier integrity, inflammatory tone, insulin sensitivity, and pancreatic *β*-cell function. Third, we discuss how integrative multi-omics (metagenomics, metatranscriptomics, proteomics, and metabolomics) can connect microbial genetic potential to *in vivo* activity and circulating metabolites, while introducing key challenges such as temporal variability, anatomical heterogeneity, and “dark matter” in gene and metabolite annotation. Fourth, strain-resolved analyses reveal that many disease-associated functions are carried by specific lineages within species, refining microbial targets and helping explain inconsistent species-level associations. Fifth, we summarize how diet shapes microbial ecology and function—supporting microbiome-informed precision nutrition—and highlight emerging evidence beyond bacteria, including viral and fungal community components. Finally, we outline translational opportunities and evidence gaps, emphasizing the need for diverse longitudinal cohorts, mechanistic validation, and well-controlled interventional trials to evaluate microbiome-directed strategies for T2D prevention and treatment.

## 1. Introduction

The global health landscape is currently dominated by a syndemic of obesity, metabolic syndrome (MetS), and type 2 diabetes (T2D), which has reached epidemic proportions and continues to escalate at an alarming rate.^1^ According to the International Diabetes Federation Diabetes Atlas, approximately 589 million adults were living with diabetes in 2024, a figure projected to surge to 853 million by 2050.^2^ T2D, characterized by progressive pancreatic *β*-cell dysfunction and often with insulin resistance,^3^ accounts for over 90% of these diabetes cases and is a leading cause of cardiovascular disease, kidney failure, and premature mortality.^4^^-^^6^ Although genetic predisposition and lifestyle factors such as physical inactivity and unhealthy dietary patterns are established drivers of this pandemic, they do not fully account for the observed inter-individual variability in disease susceptibility and progression.^7^^-^^9^ Consistent with this heterogeneity, data-driven subclassification efforts—most prominently the ANDIS approach proposed by Ahlqvist and colleagues—have identified clinically distinct adult-onset diabetes clusters (e.g., severe insulin-deficient, severe insulin-resistant, mild obesity-related, and mild age-related forms), with differing trajectories and complication risks.^10^ Emerging evidence further suggests that gut microbiome composition and/or function may differ across these subtype frameworks, although the literature remains limited and still evolving.^11^ This recognition has intensified efforts to identify additional biological mechanisms that mediate both established and emerging risk factors. In recent years, such efforts have driven a notable conceptual shift in metabolic disease research, positioning the gut microbiome as a critical environmental exposure and a central modulator of host metabolic physiology.^12^^,^^13^

Residing within the human gastrointestinal tract is a dense and dynamic ecosystem of trillions of microorganisms, including bacteria, archaea, viruses, and fungi, collectively known as the gut microbiome.^13^ Over the past two decades, sparked by seminal work from Dr. Jeffrey I. Gordon’s group and reinforced by extensive evidence from animal models and human studies, the gut microbiome has emerged as a “virtual metabolic organ”,^14^^-^^16^ encoding a broad repertoire of metabolic functions and playing a critical role in the pathogenesis of obesity, insulin resistance, and T2D.^17^^-^^19^ These works have situated the gut microbiome as mechanistically intertwined with several key pathways. First, the gut microbiome produces and transforms a broad array of bioactive metabolites that enter the circulation and function as critical signaling molecules. These include short-chain fatty acids (SCFAs), which generally promote gut barrier integrity and glucose homeostasis and are involved in the regulation of gut hormones such as glucagon-like peptide-1 (GLP-1) and peptide YY(PYY),^20^^,^^21^ as well as metabolites such as branched-chain amino acids (BCAAs) and trimethylamine *N*-oxide (TMAO), which have been implicated in promoting insulin resistance.^22^^-^^24^ Second, dysbiosis is linked to compromised intestinal barrier function—a “leaky gut” phenotype—characterized by the weakening of tight junction proteins that seal the paracellular space between enterocytes.^25^ This increased permeability facilitates the translocation of pro-inflammatory microbial components, most notably lipopolysaccharide (LPS), from the outer membrane of Gram-negative bacteria, into the host circulation.^26^ The resulting metabolic endotoxemia triggers chronic, low-grade systemic inflammation via Toll-like receptor 4 (TLR4) activation, a well-established driver of insulin resistance in peripheral tissues.^27^ The convergence of these mechanistic insights supports the gut microbiome as a plausible contributor to T2D, rather than a passive bystander.

The composition and functional activity of the gut microbiome are profoundly shaped by host diet.^28^ The intricate interplay among diet, the gut microbiome, and host physiology constitutes a critical “diet-microbiome-host” axis. This interaction is inherently bidirectional: short-term, high-intensity dietary interventions can induce rapid but transient shifts in the gut microbial communities, whereas long-term habitual dietary patterns serve as the dominant force shaping gut microbial ecology.^29^^,^^30^ Conversely, the gut microbiome actively participates in the metabolism of dietary components, modulating the bioavailability of nutrients and diet-derived bioactive compounds. Diets characteristic of Westernized societies, typically high in saturated fats, refined sugars, and processed foods, are associated with reduced microbial diversity and the proliferation of pro-inflammatory taxa, thereby exacerbating metabolic endotoxemia and insulin resistance.^31^ In contrast, dietary patterns rich in plant-based foods, such as the Mediterranean diet, promote a more diverse and functionally resilient microbiome enriched in beneficial SCFA-producing bacteria, such as *Faecalibacterium prausnitzii* and *Eubacterium rectale*, which, in turn, support gut barrier integrity and metabolic health.^32^^-^^34^

To elucidate the complexity of these interactions, the methodological landscape of microbiome and human health research has advanced rapidly over the past decade. Initial discoveries were largely driven by 16S rRNA gene sequencing, a cost-effective method for taxonomic profiling that, while foundational, is limited in its resolution, typically at the genus level, and provides no direct information on microbial function.^35^ The advent of shotgun metagenomics and metatranscriptomics represented a significant leap forward in microbiome research. These approaches enable high-resolution taxonomic characterization down to the species and even strain levels and extend beyond bacteria and fungi to achieve multi-kingdom profiling that includes viruses and other microbial domains. Moreover, they provide comprehensive, direct functional measures of community and individual taxa.^36^^,^^37^ This advancement is particularly important for metabolic disease research, as metabolic functions are often strain-specific, offering critical biological insights into pathogenic mechanisms and identifying actionable targets for microbiome-based therapeutic interventions.^36^^,^^38^ More recently, the field has entered a multi-omics era, integrating metagenomics with metatranscriptomics (transcription), proteomics (proteins), and metabolomics (metabolites).^39^^,^^40^ Such integrative, systems-level analyses are essential for constructing a holistic view of the dynamic host-microbiome interplay and for moving beyond correlation to establish mechanistic and potentially causal relationships.^41^

Given the rapid advances in this field, our aim in this review is to bring together current knowledge on how the gut microbiome shapes the development of T2D, drawing on metagenomic and emerging multi-omics studies in humans, as well as mechanistic studies. We integrate findings across taxonomic, functional, and dietary dimensions to illustrate how microbial composition, metabolic activity, and host–microbe interactions collectively shape metabolic risk. We also highlight methodological limitations, unresolved mechanistic questions, and the emerging opportunities for microbiome-directed interventions and precision nutrition. Dedicated sections examine multi-omics frameworks that reveal microbial functional networks, explore the influence of diet on microbial ecology and metabolic outcomes, and discuss the importance of strain-level diversity for understanding disease mechanisms and identifying actionable therapeutic targets.

## 2. Gut microbiome and metabolic risk: evidence from metagenomic studies

### 2.1. Altered microbial composition and taxonomic signatures in type 2 diabetes

Previous studies leveraging shotgun metagenomic sequencing have linked gut microbial community structure and specific taxa to metabolic risk factors^22^^,^^42^ and T2D.^18^^,^^43-49^ Importantly, these associations are not uniform: reported T2D microbiome signatures vary across geography and ethnicity and are strongly modified by adiposity, habitual diet, and medication exposure (especially metformin), such that species-level signals observed in one population or clinical context may not generalize directly to another. While these metagenomic associations have most consistently implicated bacterial communities, the gut microbiome also comprises archaea, viruses, fungi, and other microbial eukaryotes. At present, robust cross-cohort taxonomic “signatures” of T2D outside of bacteria are comparatively limited, reflecting both biological features (e.g., lower biomass of fungi and other eukaryotes) and methodological challenges (e.g., incomplete reference databases and heterogeneous inclusion of non-bacterial reads across pipelines).^50^ Accordingly, we focus here on the best-established bacterial taxonomic associations and discuss emerging evidence for non-bacterial domains in dedicated sections below, while highlighting multi-kingdom profiling as a key priority for future studies.

Several *Clostridium* species associated with gut inflammation, e.g., *Clostridium bolteae,*^43^^,^^44^^,^^48^^,^^51^ *Clostridium hathewayi,*^44^^,^^48^ and *Clostridium symbiosum,*^44^^,^^51^ have been consistently reported as enriched in individuals with T2D. In contrast, major butyrate-producing bacteria such as *F. prausnitzii*^43^^,^^44^^,^^48^ and *Roseburia intestinalis,*^43^^,^^44^ along with *Dorea longicatena*, a potential indole-3-acetate producer,^18^^,^^43^^,^^52^ are consistently depleted in T2D. However, these findings remain partly inconsistent, likely due to small sample sizes, heterogeneous study designs, and differences in analytical pipelines across studies. Population structure may also contribute to inconsistent findings across studies. Baseline microbiome configuration, habitual diet, medication patterns, and host genetics differ across geographic/ethnic groups, and these differences can shift both the prevalence of candidate taxa and the functional capacity encoded within a given species. Moreover, early investigations often did not adequately adjust for major confounders, including metformin use and adiposity, which can profoundly alter microbiome composition and thereby limit the interpretability and reproducibility of observed associations.^43^^,^^53^^,^^54^ These sources of heterogeneity suggest that T2D dysbiosis should be interpreted as a set of context-dependent microbial shifts rather than a single universal signature; in many settings, functional pathways and strain-level features may provide more reproducible signals across cohorts than species-level abundance alone.

To address these challenges, we recently conducted a cross-cohort analysis of 8,117 metagenomes across Asia, Europe, and North America, applying harmonized data processing and statistical modeling. This analysis identified 19 species robustly linked to T2D status. Depleted butyrate producers—*Butyrivibrio crossotus*, *Faecalibacterium prausnitzii*, *R. intestinalis*, and *Coprococcus eutactus*—emerged as consistent biomarkers of metabolic health, owing to their roles in gut barrier maintenance and anti-inflammatory signaling.^38^ Conversely, taxa *like C. bolteae*, *Bacteroides fragilis*—a producer of immunogenic fragilysin, and *Escherichia coli* were enriched in T2D guts, suggesting expansion of opportunistic, pro-inflammatory microorganisms.^18^^,^^44^ Importantly, detailed strain-resolved analyses have revealed that within-species heterogeneity often underlies inconsistent associations observed at the species level. Taking *Prevotella copri* as an example, metagenomic reconstructions and follow-up metatranscriptomic studies have shown that certain strains are enriched in fiber-rich, healthy populations and specialize in complex polysaccharide degradation, while others carry expanded clusters of branched-chain amino acid (BCAA) biosynthesis genes linked to insulin resistance.^22^^,^^55^ In addition, *P. copri* strain/subclade structure exhibits clear population patterning—non-Hispanic white participants in Europe and the United States are often dominated by clade A, whereas co-presence of multiple clades is more common in Chinese, Israeli, and US Hispanic populations—and T2D-associated functional signals can be clade-dependent (e.g., dysregulated BCAA biosynthesis in T2D concentrated within clade A in our cross-cohort analysis).^38^ Similarly, among *E. rectale* and other species, T2D-associated strains can arise in specific geographic subclades (e.g., strains from Southern China or Northern Europe) or appear across multiple populations, highlighting how population-structured strain diversity can generate inconsistent species-level signals across studies and emphasizing the value of strain-aware, cross-cohort analyses.^38^^,^^56^

### 2.2. Microbiome contributions to insulin resistance and pancreatic *β*-cell dysfunction

Insulin resistance (IR) is the central pathophysiological defect that precedes and underlies the development of T2D. It is a condition in which peripheral tissues, such as skeletal muscle, liver, and adipose tissue, fail to respond adequately to insulin.^57^ The gut microbiome is now recognized as a key environmental determinant of insulin sensitivity and *β*-cell function through multiple, interrelated mechanisms involving microbial metabolites, structural components, gut-hormone signaling, and immune-metabolic crosstalk.^58^

Bacterial cell wall components, such as LPS, peptidoglycans, and lipoteichoic acids, can initiate metabolic endotoxemia and chronic low-grade inflammation, hallmarks of IR.^26^ Translocation of LPS across a compromised intestinal barrier activates TLR4–NF-κB signaling in adipose tissue, liver, and skeletal muscle, inducing cytokines (e.g., TNF-*α*, IL-1β, IL-6) that impair insulin receptor phosphorylation and downstream signaling.^59^ In animal models, low-dose endotoxemia directly induces insulin resistance, whereas depletion of LPS-producing taxa or restoration of gut barrier integrity ameliorates metabolic inflammation.^26^ Conversely, microbe-derived SCFAs accelerate epithelial tight-junction assembly, enhance mucin secretion, and suppress pro-inflammatory cytokine release through HDAC inhibition.^60^^,^^61^ The microbiome also influences gut hormone secretion that modulates systemic glucose homeostasis. SCFAs such as butyrate and propionate stimulate enteroendocrine L-cells via G-protein-coupled receptors (GPR41/43) to enhance secretion of GLP-1 and PYY, which collectively increase insulin secretion, improve peripheral insulin sensitivity, and promote satiety.^62^^-^^64^ Beyond SCFAs and bile acids, several additional microbiota-derived metabolites have been recently linked to T2D-relevant pathophysiology. A prominent example is imidazole propionate, a gut-derived histidine metabolite that impairs insulin signaling through activation of the p38γ–p62–mTORC1 axis and is elevated in individuals with T2D; subsequent work suggests it can also attenuate glucose-lowering responses to metformin, highlighting clinically relevant microbe–drug interactions.^65^ Microbial tryptophan-derived indole metabolites also appear relevant: circulating indole-3-propionic acid (IPA) has been associated with lower risk of incident T2D and preservation of insulin secretion capacity in human longitudinal data, and broader indole derivatives are increasingly recognized as immunometabolic modulators (e.g., barrier and inflammatory signaling).^66^ In addition, microbial phenolic metabolites from aromatic amino acids—including *p*-cresol and its conjugate *p*-cresyl sulfate—have been implicated in host metabolic regulation, with experimental evidence that *p*-cresol can suppress gut hormone transcriptional programs and that *p*-cresyl sulfate can promote insulin resistance and inflammatory/oxidative stress phenotypes *in vivo*.^67^ Finally, dysbiotic microbiota can generate endogenous ethanol, which has been linked to hepatic metabolic injury in NAFLD/NASH—conditions tightly coupled to insulin resistance and highly prevalent in T2D—suggesting a plausible liver-centric pathway through which microbial metabolism may exacerbate cardiometabolic risk.^68^

In human metagenomic studies, at the microbial composition level, increased abundance of *P. copri* and *Bacteroides vulgatus* has been consistently identified as hallmarks of insulin resistance. This association is strongly linked to the microbial metabolism of BCAAs, a key pathway implicated in IR.^22^ Likewise, members of the *Lachnospiraceae* family, particularly species from the genera *Dorea* and *Blautia*, are enriched in IR individuals and are associated with an altered capacity for carbohydrate metabolism that promotes inflammation.^69^ In contrast, insulin-sensitive individuals tend to harbor *Alistipes indistinctus* and certain *Bacteroides* species that counteract the accumulation of pro-inflammatory metabolites derived from carbohydrates,^58^ together with a higher abundance of SCFA-producing bacteria, such as *F. prausnitzii*, which are consistently associated with improved insulin sensitivity and glucose tolerance.^69^ Emerging evidence also implicates the microbiome in pancreatic *β*-cell dysfunction, a key event in T2D progression. Microbial metabolites and structural components can directly or indirectly affect *β*-cell viability and insulin secretory capacity. Circulating LPS and inflammatory cytokines impair *β*-cell insulin secretion and promote apoptosis through NF-κB activation and endoplasmic reticulum stress.^70^ Conversely, SCFAs and secondary bile acids can enhance insulin secretion by activating GPR43 and TGR5 receptors on *β*-cells, respectively.^71^ Furthermore, microbial metabolites such as TMAO and phenylacetylglutamine have been shown to disrupt calcium signaling and mitochondrial function in *β*-cells, contributing to impaired insulin release and *β*-cell death (Table 1).^70^

**Table 1.** Microbiota-derived metabolites implicated in type 2 diabetes (T2D) pathophysiology and host targets.

Metabolite	Major microbial origin/pathway	Key host targets/mechanisms	T2D-relevant phenotypes
SCFAs (butyrate, propionate, acetate)	Fermentation of dietary fiber by saccharolytic bacteria	↑ gut barrier (tight junctions, mucin); anti-inflammatory signaling; HDAC inhibition (butyrate); ↑ GLP-1/PYY via GPR41/43	Improved insulin sensitivity; ↓ endotoxemia/inflammation; improved glycemic control
Secondary bile acids	Microbial bile acid transformations (e.g., BSH/HSDH)	Modulate FXR/TGR5; affect hepatic glucose/lipid metabolism; influence incretin/endocrine signaling	Links microbial activity to hepatic/pancreatic glucose regulation; impacts insulin sensitivity
BCAA-related microbial functions/metabolites	Microbial BCAA biosynthesis capacity (strain- and pathway-dependent)	Associated with insulin resistance signatures; may reflect shift toward amino-acid metabolism & mitochondrial stress pathways	Insulin resistance; metabolic inflammation
Imidazole propionate	Microbial histidine metabolism	Impairs insulin signaling via p38γ–p62–mTORC1; may blunt metformin response (microbe–drug interaction)	Insulin resistance; reduced drug responsiveness
Indole-3-propionic acid (IPA) *(tryptophan-derived)*	Microbial tryptophan metabolism → indole derivatives	Barrier/anti-inflammatory effects; linked to *β*-cell preservation; immune–metabolic modulation	Lower incident T2D risk; preserved insulin secretion capacity (human longitudinal data)
Other tryptophan-derived indoles (e.g., indole/IAA derivatives)	Microbial tryptophan metabolism	Immune–metabolic regulation; barrier integrity; inflammatory tone	Potential influences on insulin resistance/inflammation
*p*-cresol/*p*-cresyl sulfate (phenolic metabolites)	Microbial aromatic amino acid metabolism → *p*-cresol; host conjugation (sulfation) → *p*-cresyl sulfate	*p*-cresol: may perturb epithelial/endocrine signaling; *p*-cresyl sulfate: pro-oxidative/pro-inflammatory signaling; associated with insulin resistance–related pathways	Insulin resistance signatures; inflammation/oxidative stress; altered gut–endocrine axis
Endogenous ethanol	Microbial fermentation (dysbiosis-associated ethanol producers)	Hepatic oxidative stress; links to NAFLD/NASH pathways tied to insulin resistance	Liver-centric metabolic injury; worsened cardiometabolic risk
TMAO	Microbial choline/carnitine → TMA; host FMO3 → TMAO	Pro-inflammatory cardiometabolic signaling; vascular dysfunction pathways; may relate to *β*-cell stress in some studies	Insulin resistance/β-cell stress pathways; cardiometabolic risk
Phenylacetylglutamine	Microbial aromatic amino acid metabolism (phenylalanine-related)	Alters host signaling; associated with cardiometabolic pathways	Impaired insulin release; *β*-cell stress

Abbreviations: SCFAs, short-chain fatty acids; BSH, bile salt hydrolase; HSDH, hydroxysteroid dehydrogenase; FXR, farnesoid X receptor; TGR5, G-protein–coupled bile acid receptor; PYY, peptide YY; GLP-1, glucagon-like peptide-1; IAA, indole-3-acetic acid; TMA, trimethylamine; TMAO, trimethylamine-N-oxide; FMO3, flavin-containing monooxygenase 3; NAFLD/NASH, nonalcoholic fatty liver disease/steatohepatitis.

### 2.3. The role of virome

The human virome, encompassing both eukaryotic viruses and bacteriophages, is increasingly recognized as an integral component of host–microbiome interactions.^72^ Advances in sequencing and computational tools now enable comprehensive multi-kingdom profiling of viral communities. Reflecting this maturation of the field, the NIH has launched the Human Virome Program, within which our group co-leads a Virome Characterization Center. Among eukaryotic viruses, several—including herpesviruses,^73^ polyomaviruses,^74^ and HTLV-1^75^—can establish persistent infections with periodic reactivation, promoting chronic inflammation and immune dysregulation implicated in T2D pathogenesis.^76^ Experimental data further show that viral RNA can impair adipocyte insulin signaling by activating the IRF3 pathway,^77^ while enteroviruses can infect pancreatic *β*-cells and adenovirus 36 can increase adiposity in animal models.^78^ Beyond eukaryotic viruses, bacteriophages profoundly influence gut bacterial community composition and function.^79^^,^^80^ Phages targeting beneficial taxa such as butyrate producers may promote insulin resistance, whereas others suppress pro-inflammatory species.^81^ Elevated levels of *Caudovirales* and *crAssphage* have been linked to metabolic syndrome in humans.^82^ Phages also mediate horizontal gene transfer, including genes for virulence and carbohydrate metabolism. Our recent study demonstrated that phage-mediated gene transfer can generate strain-specific metabolic capabilities linked to T2D risk.^38^ In mice, fecal virome transplantation from lean donors improved glucose tolerance in obese diabetic hosts, supporting a potentially causal role for the virome in metabolic regulation.^83^^,^^84^ In that study, metabolic improvement coincided with measurable shifts in both the recipient virome (differentially abundant viral contigs/viral taxa and predicted bacterial hosts) and the bacteriome, consistent with phage-driven remodeling of bacterial community structure and function.^84^ These data do not yet implicate a single causal virus; instead, they support a community-level mechanism in which phage–bacteria interactions (and potentially downstream immune effects) mediate metabolic outcomes—an interpretation aligned with human virome studies that emphasize disrupted virus–bacteria interaction networks in T2D.^84^ However, human interventional evidence remains limited: a double-blind randomized fecal filtrate transplantation trial in metabolic syndrome showed feasibility and short-term phageome changes but did not demonstrate significant overall improvement in glucose metabolism over short follow-up, underscoring that this area is still emerging and hypothesis-generating.^85^

### 2.4. The role of the mycobiome (fungome)

The gut ecosystem also includes commensal fungi (the gut mycobiome, or “fungome”), which—despite lower biomass than bacteria—may exert disproportionate effects on host immunity and metabolic physiology through cross-kingdom interactions and fungal-derived structural components and metabolites. Available human evidence in T2D remains limited and somewhat heterogeneous, but several reports suggest altered fungal community composition and enrichment of specific taxa (e.g., *Candida*) in T2D; importantly, recent work highlights that antidiabetic therapies can confound associations between gut fungi and T2D, underscoring the need to account for medication use and other key host factors in mycobiome analyses.^86^ Mechanistically, fungal cell wall components such as *β*-glucans can activate pattern-recognition pathways (notably CLEC7A/Dectin-1 signaling) that promote inflammatory responses relevant to insulin resistance. In mouse models, abnormal expansion of gut fungi (including *Candida albicans* or *C. albicans*–associated gut fungal blooms) has been shown to exacerbate metabolic inflammation and insulin resistance via Dectin-1–dependent pathways, providing proof-of-concept that fungi can contribute to metabolic dysregulation.^87^ Additional experimental evidence further supports a role for gut fungus–host immune signaling (e.g., CLEC7A/Dectin-1) in diet-induced metabolic phenotypes, including lipid deposition and obesity-related metabolic dysfunction.^88^ Collectively, these findings motivate more systematic multi-kingdom profiling (bacteriome–virome–mycobiome) and integrative analyses of fungal–bacterial interactions in well-powered, longitudinal cohorts and intervention studies to clarify whether mycobiome features serve as subtype-specific biomarkers, causal drivers, or treatment-responsive mediators in the microbiome–T2D axis.^89^

### 2.5. Functional reprogramming of the gut microbiome in type 2 diabetes

Beyond compositional alterations, T2D is characterized by a functional reprogramming of the gut microbiome, which collectively reshapes host metabolism through its vast network of biochemical activities.^90^ Metagenomic and metabolomic analyses reveal that microbial communities in T2D exhibit a coordinated shift toward enhanced amino acid and carbohydrate fermentation, diminished SCFA and secondary bile acid production, and increased generation of proinflammatory and insulin-desensitizing metabolites.^22^^,^^90^^,^^91^ These functional perturbations converge on a limited number of host pathways that regulate insulin sensitivity, lipid and glucose homeostasis, and inflammation.^22^^,^^90^ Rather than single metabolites acting in isolation, these alterations constitute a dynamic metabolic network in which multiple microbial products interact and reinforce each other’s effects. Perturbation in one pathway—for example, reduced short-chain fatty acid production—can amplify dysfunction in others, such as impaired gut barrier integrity, systemic inflammation, and amino acid–driven activation of nutrient-sensing pathways like mTOR.^22^^,^^90^ Likewise, microbial remodeling of bile acid and choline metabolism modifies host endocrine signaling (e.g., GLP-1, FXR–FGF15/19, TGR5), thereby linking intestinal microbial activity directly to hepatic and pancreatic function.^90^^,^^92^ Such interdependence highlights that T2D-associated dysbiosis represents a systems-level metabolic state rather than a sum of isolated metabolite changes. Collectively, these findings support a model in which microbial metabolic networks act as an intermediary layer between environmental exposures (e.g., diet, drugs) and host metabolic responses.^22^^,^^90^ A well-established example is metformin, the first-line pharmacotherapy for T2D, which consistently alters gut microbial composition and function and can confound observational microbiome–T2D associations.^93^ Importantly, mechanistic work using a host–microbe–drug–nutrient screening framework identified bacterial effectors of metformin therapy and implicated specific microbial metabolic pathways, including agmatine-related metabolism, as functional mediators of drug response.^94^ This illustrates how common antidiabetic medications can influence the microbiome–host metabolic axis at the level of microbial functions, not only community composition.^38^^,^^94^ These pharmacotherapy effects underscore that medication exposure is a major effect modifier and should be modeled explicitly when interpreting microbial functional signatures of T2D. Beyond metformin, other glucose-lowering therapies—particularly GLP-1 receptor agonists (GLP-1RAs)—may influence the gut ecosystem and intersect with bile acid signaling, with implications for interpreting T2D microbiome signatures in treated populations. GLP-1RAs alter gastrointestinal physiology (e.g., gastric emptying and intestinal transit) and have been reported to modulate gut microbial composition in animal models and in some human studies, with effects that may be modest over short exposure but more apparent with longer treatment durations.^95^ Mechanistically, bile acids provide a key link between microbiome function and incretin biology: microbial bile acid transformations shape activation of FXR and TGR5, which regulate glucose homeostasis and can influence GLP-1 secretion; conversely, incretin-based therapy may shift bile acid pools and downstream signaling pathways.^96^ Integrative multi-omics approaches are beginning to reveal the architecture of this gut–metabolism interface, yet most current frameworks are derived from a limited set of well-characterized metabolites.^22^^,^^91^ The gut ecosystem likely harbors a vastly larger repertoire of unexplored bioactive molecules—including peptides, cofactors, xenobiotic derivatives, and strain-specific products—that remain to be mapped.^90^ Expanding this biochemical space will be critical to refining our understanding of how microbial metabolism drives insulin resistance and the pathogenesis of T2D.

**Figure 1.**
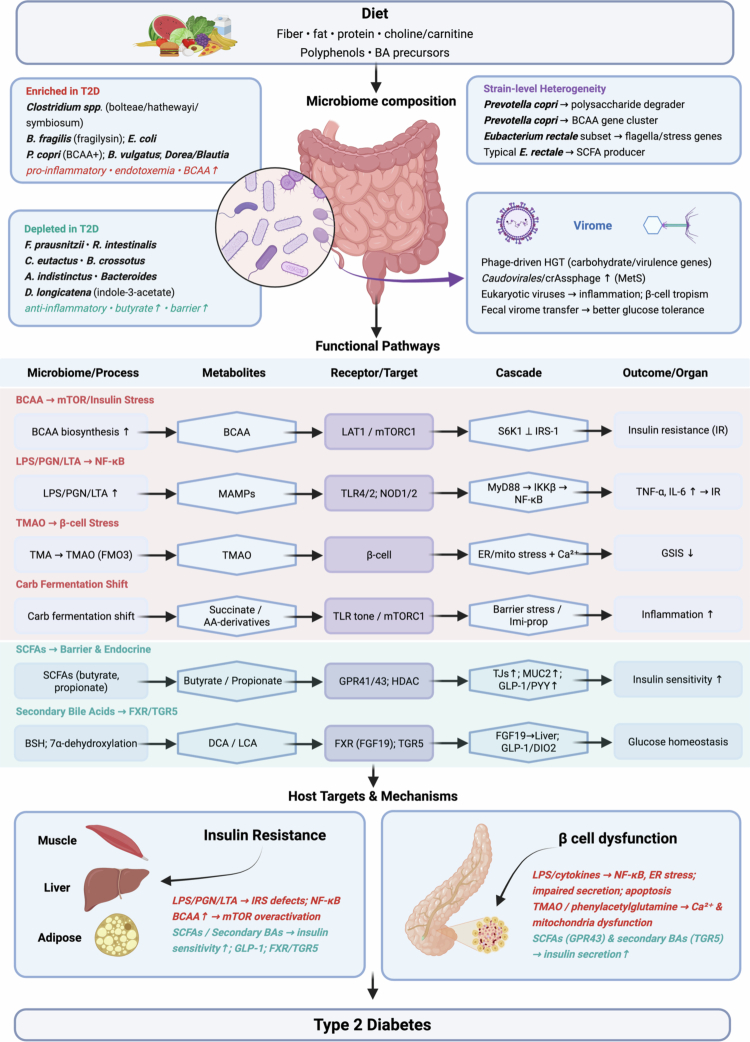
Diet–microbiome interactions and microbial contributors to type 2 diabetes. Dietary components—including fiber, fats, proteins, and polyphenols—profoundly shape gut microbial composition and function. These microbial shifts play a central role in T2D pathogenesis, as illustrated by numerous taxa consistently linked to metabolic health or disease. The field’s transition from species-level surveys to high-resolution, strain-resolved profiling has revealed that many disease-associated functions are strain-specific, a layer of biological variation missed by earlier approaches. Beyond bacteria, emerging studies of archaeal and viral communities, including the gut virome, highlight additional—and largely unexplored—microbial pathways contributing to metabolic dysfunction. Representative taxa enriched in T2D (red) include *Clostridium spp.* (*C. bolteae, C. hathewayi, C. symbiosum*), *Bacteroides fragilis* (fragilysin+), *Escherichia coli*, *Prevotella copri* (BCAA+ strains), *Bacteroides vulgatus*, and *Dorea/Blautia* (*Lachnospiraceae*), which are linked to greater BCAA biosynthesis, LPS/PGN/LTA load, and a carbohydrate fermentation shift toward pro-inflammatory metabolites. Depleted in T2D (green) are SCFA-producing/anti-inflammatory taxa (e.g., *Faecalibacterium prausnitzii*, *Roseburia intestinalis*, *Butyrivibrio crossotus*, *Coprococcus eutactus*, *Alistipes indistinctus*, selected *Bacteroides*, and *Dorea longicatena*), supporting barrier function and anti-inflammation. Functional modules converge on host targets: SCFAs tighten epithelial junctions, increase mucin, inhibit HDACs, and stimulate GLP-1/PYY (via GPR41/43), improving insulin sensitivity and *β*-cell function; secondary bile acids signal via FXR/TGR5. Conversely, LPS/PGN/LTA activate TLR4–NF-κB and cytokines (TNF-*α*, IL-1β, IL-6), while TMAO (from hepatic FMO3) perturbs *β*-cell Ca²⁺ handling and mitochondria. Strain-level heterogeneity (e.g., BCAA-rich vs fiber-degrading *P. copri*) and virome effects (phage-mediated HGT; *Caudovirales*/crAssphage shifts; eukaryotic viruses) reprogram community function. These interactions promote insulin resistance (muscle/liver/adipose) and *β*-cell dysfunction, culminating in T2D. This illustration was created with BioRender.com. Abbreviations: T2D, type 2 diabetes; SCFA, short-chain fatty acid; BCAA, branched-chain amino acid; LPS, lipopolysaccharide; PGN, peptidoglycan; LTA, lipoteichoic acid; TMA, trimethylamine; TMAO, trimethylamine-*N*-oxide; BA, bile acid; GLP-1, glucagon-like peptide-1; PYY, peptide YY; GPR41/43, SCFA receptors; HGT, horizontal gene transfer; IR, insulin resistance; ER, endoplasmic reticulum; NF-κB, nuclear factor kappa-B; IRS, insulin receptor substrate; FMO3, flavin-containing monooxygenase 3; GSIS, glucose-stimulated insulin secretion; BSH, bile salt hydrolase; DCA, deoxycholic acid; LCA, lithocholic acid; FXR, farnesoid X receptor; TGR5, G-protein–coupled bile acid receptor.

Despite remarkable advances, current human microbiome studies still face conceptual and methodological challenges that limit their interpretability and translational potential. Most human studies remain cross-sectional and observational, which, although useful for identifying associations, cannot establish causality. Longitudinal and interventional studies, along with mechanistic validation in model systems, are urgently needed to disentangle cause from consequence and identify modifiable microbial drivers of metabolic dysfunction.^97^ Moreover, host and environmental factors—including medication use, diet, genetics, age, sex, body mass index, and geography—exert powerful influences on microbiome composition.^33^^,^^43^^,^^54^^,^^93^ For example, metformin profoundly alters the gut ecosystem, notably increasing *Akkermansia muciniphila* and *E. coli*, potentially confounding T2D associations unless treatment-naïve populations are studied.^43^^,^^93^ Early reliance on 16S rRNA gene sequencing imposed limitations on taxonomic resolution and functional inference, while PCR biases further risked misleading conclusions.^97^ Although ecological metrics such as alpha- and beta-diversity provide useful community-level summaries, they often lack direct biomedical relevance and may conceal critical changes in specific taxa or pathways.^98^^,^^99^ The field must also guard against underpowered studies that can detect only large effect sizes and against “p-hacking” through indiscriminate testing of multiple diversity metrics.^100^ Moving forward, progress in this field will depend on large-scale, prospectively designed, diverse human cohorts and consortium-scale study of multiple cohorts with harmonized data processing and analysis. In summary, large-scale metagenomic investigations have begun to delineate a coherent taxonomic and functional signature of the T2D gut microbiome. Yet translating these associations into mechanistic insights and clinical applications demands rigorous study design, careful control of confounders, deeper strain-level and functional analyses, and integration with longitudinal and interventional research (Figure 1).

## 3. Multi-omics approaches to gut microbial composition and function

### 3.1. Integrating metagenomics, metatranscriptomics, and metabolomics

Understanding the complex interplay between the gut microbiota and host metabolism in T2D requires moving beyond single-omics analyses toward a systems biology perspective. Microbial communities exhibit extensive functional redundancy, in which different taxa can perform similar biochemical reactions through distinct genetic repertoire.^101^ Therefore, resolving microbial contributions to metabolic phenotypes necessitates characterizing not only “who is there” but also “what they are doing”, encompassing genetic potential, transcriptional activity, and metabolic output.

Metagenomics provides a foundation for functional inference by cataloging microbial genes within communities and specific taxa, offering both taxonomic profiles and an inventory of enzymatic potential.^39^ However, genomic presence alone does not guarantee activity: many encoded genes may remain transcriptionally silent or conditionally expressed. This limitation underscores the importance of metatranscriptomics, which captures microbial transcription and thus provides a snapshot of real-time microbial activity under specific dietary or physiological states.^102^ Specifically, metatranscriptomics quantifies community-wide gene expression (RNA), thereby complementing metagenomics (DNA-based functional potential) by distinguishing which microbial pathways are actively engaged *in vivo* at the time of sampling.^103^ Recent large-scale human fecal metatranscriptome resources have further improved the feasibility of linking microbial transcriptional programs to host phenotypes across diverse populations, helping to move beyond inference from gene content alone.^103^ For example, in a controlled dietary study with continuous glucose monitoring, metatranscriptomic activity features contributed significantly to predicting inter-individual variability in postprandial glycemic response, with specific microbial functional programs (e.g., fucose metabolism and indoleacetate-related pathways) emerging as activity-level correlates of glycemic control.^104^ In one treatment-naïve Chinese cohort spanning normal glucose tolerance, prediabetes, and T2D, combined metagenomics–metaproteomics revealed that treatment-naïve T2D is characterized by lower fecal host antimicrobial peptides (e.g., defensin-5, neutrophil defensin-1, lysozyme C) and lower digestive proteases/lipases (trypsin/chymotrypsin and precursors), alongside higher amylase (AMY1), and that microbial protein signatures differed by disease stage (e.g., *Bacteroides* meta-proteins enriched in treatment-naïve T2D).^49^ In our work, integrating metagenomic and metatranscriptomic datasets revealed that dietary fiber and plant food intake are tightly linked to both the functional potential and transcription of pectin-degrading enzymes, offering stronger evidence for diet-driven microbial functions.^33^ Despite its power, metatranscriptomic profiling is inherently sensitive to transient environmental cues, reflecting the highly dynamic nature of microbial gene expression. Metabolomics adds a complementary layer by quantifying small-molecule metabolites, the biochemical end products of microbial and host activities, thereby connecting microbial functions to systemic metabolism.^105^ Notably, several recent human multi-omics studies integrating gut metagenomics with metatranscriptomics and/or fecal/plasma metabolomics have shown that microbial “activity” signals and metabolite readouts are associated with T2D-related pathogenic pathways beyond what taxonomic profiles alone reveal, thereby strengthening mechanistic interpretation.^49^^,^^106^^,^^107^ For example, in our study, we identified both metagenomic functional pathways and plasma metabolites related to secondary bile acid metabolism that were associated with changes in body weight and serum lipid profiles.^107^ Additionally, the Integrative Human Microbiome Project, which combined metagenomics and metatranscriptomics, revealed a shift toward aerotolerant, pro-inflammatory ecological functions and altered community activity in the context of inflammatory bowel disease (IBD). Although these findings pertain primarily to IBD, they carry potential implications for peripheral systemic inflammation, a recognized pathway contributing to T2D pathogenesis.^108^ Untargeted metabolomics has identified circulating amino acids, lipids, and other metabolites that are prospectively associated with T2D risk, serving as mechanistic biomarkers that link gut microbial metabolism to host insulin sensitivity and glucose regulation. Gut microbes modify primary bile acids into diverse secondary forms that act as potent signaling molecules interacting with host receptors such as FXR and TGR5, thereby influencing glucose and lipid homeostasis.^71^ The activity of bacterial bile salt hydrolases (BSHs) and hydroxysteroid dehydrogenases (HSDHs) shapes these transformations, and their abundance has been associated with metabolic phenotypes including T2D.^109^ Our recent multi-omics analysis in the DIRECT-PLUS trial, combining fecal BAs profiling by UPLC-MS, shotgun metagenomics, and cardiometabolic phenotyping, demonstrated that baseline microbial BA metabolism predicts and modulates response to Mediterranean diet interventions in humans.^107^ Specific fecal BAs (e.g., lithocholic acid, taurolithocholic acid-3-sulfate) were prospectively associated with BMI and lipid changes over 18 months, and these associations were stronger in individuals harboring BA-metabolizing taxa such as *Ruminococcus gnavus* and *Bacteroides spp*.^107^ Strain-specific bile salt hydrolase and hydroxysteroid dehydrogenase activities markedly shaped the secondary BA pool and, in turn, influenced the magnitude of dietary benefits on adiposity and lipid profiles.^107^ These results from multi-omics studies indicate the potential of fecal BA signatures and their microbial drivers as predictive biomarkers to tailor precision dietary strategies for cardiometabolic health.

### 3.2. Temporal dynamics and anatomical heterogeneity in T2D microbiome studies

Despite such advances, key challenges remain. A defining yet challenging feature of the gut microbiome is its highly dynamic nature, which becomes particularly apparent when comparing different molecular layers. While the metagenome remains relatively stable within an individual over months to years, reflecting a person’s long-term microbial potential, the metatranscriptome is markedly more variable.^103^ Short-term fluctuations in microbial gene expression within the same individual can equal or even exceed the variability observed across different individuals at a single time point.^101^ This is particularly relevant for T2D, where first-line therapies (e.g., metformin) and dietary modifications can induce rapid and substantial microbial shifts, complicating interpretation of case–control differences if treatment exposure is not carefully modeled. Moreover, T2D progression is gradual, and microbial functions that contribute to insulin resistance may fluctuate over time with weight change, glycemic control, and inflammation, increasing the risk of reverse causation in cross-sectional comparisons. Longitudinal sampling anchored to clinically meaningful windows (e.g., prediabetes onset, medication initiation, or dietary intervention periods) is therefore critical for attributing microbial functional changes to T2D pathophysiology. This stability–variability paradox implies that a single fecal sample can reliably characterize an individual’s baseline genetic potential but cannot capture transient functional activities that shift rapidly in response to diet, medication, and other environmental stimuli.^101^ Consequently, cross-sectional studies based on one-time sampling—still the predominant design in human microbiome research—risk drawing incomplete conclusions about which microbial pathways are truly active and mechanistically linked to disease processes such as T2D.^97^ Because the transition from normal glucose tolerance to prediabetes and overt T2D unfolds gradually over years, longitudinal sampling is essential to track the temporal dynamics of microbial functions that may influence this progression.^49^ Compounding this complexity, the gastrointestinal tract is not a homogeneous environment but rather a long, anatomically diverse system. Distinct regions—from the stomach to the small and large intestines—differ in pH, oxygen tension, nutrient availability, and transit time, creating specialized ecological niches that harbor distinct microbial communities and metabolic functions.^110^^,^^111^ Moreover, microbial ecosystems in extraintestinal sites, such as the oral and respiratory tracts, can contribute to systemic inflammation and metabolic dysfunction. Most human microbiome studies rely on fecal samples because they are accessible and noninvasive, yet these samples primarily represent the distal colon. As a result, they may not fully reflect the composition or activity of microbes residing in the proximal colon or small intestine, key regions for nutrient absorption and enteroendocrine signaling, or in other body niches.^111^ This anatomical limitation is especially important in T2D because proximal gut processes—including nutrient sensing and incretin signaling (GLP-1/PYY) as well as bile acid–FXR/TGR5 pathways—are central to glucose homeostasis, yet may not be adequately captured by stool-based profiling alone.^112^ Finally, even when specific microbial metabolites are detected in circulation, distinguishing whether they originate from host metabolism, dietary sources, or microbial synthesis remains a major analytical challenge.^39^

A further T2D-specific constraint is that a substantial fraction of microbial genes and metabolite features remains unannotated, limiting our ability to map microbial pathways onto known mechanisms of insulin resistance and *β*-cell dysfunction.^113^ In practice, this “dark matter” likely includes uncharacterized enzymes and metabolites involved in xenobiotic/drug metabolism, bile acid transformations, and amino-acid–derived signaling compounds that are repeatedly implicated in metabolic phenotypes.^114^ Improving functional annotation and experimentally validating candidate pathways will be essential for translating multi-omics associations into actionable targets for microbiome-directed interventions in T2D.

### 3.3. Computational advances and challenges in high-dimensional multi-omics integration

Finally, as multi-omics datasets grow in scale, dimensionality, and complexity, advanced computational approaches are becoming indispensable. Beyond conventional machine learning (ML) models, which have been widely applied to identify disease-associated biomarkers and predictive signatures, deep learning (DL) frameworks and modern statistical methods that account for high-dimensional, sparse, and compositional data structures are increasingly critical.^115^^,^^116^ Deep neural networks, graph-based models, and representation learning approaches can capture nonlinear interactions and hierarchical dependencies between microbial, metabolic, and host features, while Bayesian and multivariate regularization techniques provide interpretable statistical inference in high-dimensional settings.^41^ Nonetheless, these models are prone to overfitting, a lack of interpretability, and poor cross-cohort generalizability if applied without rigorous validation. Achieving robust and reproducible results will require large, harmonized, and longitudinal datasets, careful model calibration, and transparent benchmarking across populations.

Collectively, these methodological and analytical advances underscore that the path from microbiome associations to mechanistic and translational insights in T2D depends on high-resolution, longitudinal, and functionally integrated approaches. Continued integration of multi-omics data, spanning genomes, transcripts, metabolites, and host phenotypes, promises to reveal a more complete and causally coherent view of how the gut microbiome modulates metabolic health.

## 4. Diet, gut microbiome, and metabolic disease risk

### 4.1. The diet–microbiome–host axis in metabolic health

The complex interplay among habitual dietary intake, the gut microbiome, and host metabolic health is a rapidly advancing field reshaping our understanding of metabolic disease. Long-term dietary patterns are among the strongest determinants of gut microbial composition and function^117^; sustained shifts in diet can remodel microbial communities in ways that either promote our metabolic resilience or increase susceptibility to metabolic dysfunction.^118^ Conversely, the gut microbiome fundamentally contributes to host physiology by metabolizing dietary components humans cannot digest, producing a diverse array of bioactive compounds that exert systemic effects on inflammation, insulin sensitivity, and cardiometabolic pathways. This bidirectional, diet–microbiome–host axis has profound implications for the development and progression of metabolic disorders such as obesity, T2D, and cardiovascular disease.^118^ The fundamental components of our diet exert distinct and powerful influences on the composition and functional capacity of our gut microbiome.

### 4.2. Dietary fiber and microbial SCFA production

Dietary fiber—structurally diverse, plant-derived carbohydrates that escape digestion by human enzymes—is a cornerstone dietary exposure in T2D research in large part because it is a primary substrate for microbial fermentation and SCFA production.^119^ Upon reaching the colon, fiber is metabolized by specialized taxa into short-chain fatty acids (SCFAs), primarily butyrate, propionate, and acetate, which act as signaling molecules that influence glucose homeostasis through effects on the intestinal barrier, immune tone, and enteroendocrine function.^120^ Butyrate is the principal energy source for colonocytes and supports mucosal integrity and anti-inflammatory signaling, whereas propionate and acetate can modulate hepatic metabolism and systemic energy balance. Importantly for T2D pathophysiology, SCFAs also promote secretion of gut hormones such as GLP-1 and PYY and can thereby influence insulin secretion, satiety, and peripheral insulin sensitivity.^64^

Human intervention evidence supports these mechanisms in the context of T2D. In a landmark dietary intervention among individuals with T2D, a high–diversity fiber diet selectively enriched SCFA-producing organisms and improved glycemic control and cardiometabolic risk factors, providing proof-of-concept that targeting fiber-responsive microbial consortia can improve T2D-relevant outcomes.^119^ Consistent with this, meta-analyses of randomized trials show that adjunctive viscous/soluble fiber supplementation improves conventional glycemic markers in T2D, including reductions in HbA1c and fasting glucose.^121^

Beyond fiber itself, evidence linking SCFAs to insulin sensitivity is accumulating. A systematic review and meta-analysis of intervention studies reported that higher post-intervention SCFA levels are associated with lower fasting insulin, supporting a beneficial relationship between SCFA generation and insulin sensitivity.^122^ Complementary human studies also suggest that circulating (rather than fecal) SCFA levels are associated with GLP-1 concentrations and measures of insulin sensitivity, underscoring the importance of integrating stool-based multi-omics with circulating metabolite readouts when inferring mechanism in T2D.^123^ Collectively, these clinical and mechanistic data support a model in which higher fermentable fiber intake can shift microbial community function toward SCFA production and downstream endocrine–immune pathways that improve insulin resistance and metabolic inflammation, while also highlighting the need for well-powered trials that jointly quantify microbial activity and host metabolic endpoints to establish causal pathways in T2D.^119^

At the community level, fermentable fiber supports a set of primary degraders and cross-feeding networks that enrich SCFA production; accordingly, key fiber-degrading taxa (e.g., *Bifidobacterium* and selected lactic acid bacteria) have been associated with improved glycemic and inflammatory profiles in human studies.^124^^,^^125^ In contrast, the chronically low fiber content of Western dietary patterns reduces fermentable substrate availability, diminishing SCFA output and favoring expansion of pro-inflammatory/pathobiont taxa—changes consistent with the dysbiotic signatures repeatedly observed in T2D.^126^ Epidemiologic comparisons further suggest that fiber-deficient dietary patterns correlate with reduced abundance of beneficial fermenters, contraction of microbial functional diversity, and higher prevalence of metabolic syndrome and T2D.^120^^,^^127^

### 4.3. Polyphenols and microbial biotransformation

Polyphenols constitute a vast and structurally diverse family of compounds found in plant-based foods such as fruits, vegetables, tea, and red wine, and have been increasingly implicated in T2D prevention and metabolic health.^128^ In large prospective cohorts and meta-analyses, higher intake of total flavonoids and specific subclasses—particularly anthocyanins/anthocyanin-rich fruits—has been associated with a lower risk of incident T2D, supporting relevance beyond general antioxidant effects.^129^ In addition, meta-analyses of controlled trials indicate that flavonoid supplementation can modestly improve glycemic biomarkers (e.g., fasting glucose, HbA1c) and insulin sensitivity, though effects vary by compound class, dose, and study population.^130^

Only a small fraction of ingested polyphenols is absorbed in the small intestine; the majority reaches the colon intact, where they undergo extensive biotransformation by the gut microbes into smaller, more bioavailable and often more potent metabolites.^128^ Polyphenols can directly modulate the gut microbial ecology by selectively inhibiting pathogenic bacteria while promoting the proliferation of beneficial species. For instance, tea catechins such as epigallocatechin-3-gallate (EGCG) and gallocatechin gallate (GCG), cocoa flavanols (catechin, epicatechin), and red wine grape polyphenols have been shown to suppress *Clostridium histolyticum* while promoting the growth of *Bifidobacterium* and *Lactobacillus spp.*^131^^-^^134^ In turn, the microbial metabolism of polyphenols gives rise to a host of bioactive compounds. For example, ellagitannins—abundant in pomegranates, berries, and nuts—are hydrolyzed to ellagic acid and subsequently converted into urolithins by gut microbes^135^ into urolithins, which possess anti-inflammatory and cardiometabolic protective properties.^136^ Together, these findings support incorporating polyphenols into a diet–microbiome–host framework for T2D, while underscoring the need for mechanistic human trials that jointly quantify polyphenol metabolites, microbiome function, and glycemic endpoints.

### 4.4. Coffee bioactives and microbial metabolism

Coffee is a widely consumed beverage containing multiple bioactives, including caffeine, polyphenolic chlorogenic acids, and melanoidins, and prospective evidence consistently supports an inverse association between coffee consumption and incident T2D.^137^^-^^141^ Emerging evidence indicates that coffee consumption beneficially modulates the gut microbiome.^140^^,^^142^ Chlorogenic acids are candidate mediators of these benefits, and their biotransformation yields metabolites including quinic acid; in a large multi-cohort multi-omics study, coffee intake was strongly associated with higher *Lawsonibacter asaccharolyticus* abundance, and plasma metabolomics showed quinic acid (and correlated derivatives) enriched among coffee drinkers in a manner linked to *L. asaccharolyticus.*^143^ In this context, quinic acid should be interpreted primarily as a coffee-derived metabolite of coffee biotransformation rather than as an established causal mediator of T2D risk; direct human evidence that quinic acid reduces T2D incidence is currently limited.^143^ Nevertheless, experimental work suggests biological plausibility: quinic acid can enhance glucose-stimulated insulin secretion and improve glucose tolerance in mouse models, consistent with potential effects on *β*-cell function.^144^ Regarding whether *L. asaccharolyticus* differs between healthy individuals and those with T2D, the same multi-cohort analysis reported high prevalence in both healthy and non-healthy Westernized populations and did not observe a strong disease association in meta-analysed public case–control metagenomic datasets across multiple conditions (including cardiometabolic diseases), highlighting that T2D-specific differences are not yet consistently established and may depend on background coffee exposure.^143^

### 4.5. Fermented dairy and probiotics

Fermented dairy refers to dairy foods produced by controlled fermentation with starter cultures (e.g., yogurt, kefir, and other cultured milks), which can deliver live microbial communities and fermentation-derived metabolites within a food matrix.^145^ In prospective cohort studies and updated meta-analyses, yogurt intake has been associated with a lower risk of incident T2D, supporting relevance of fermented dairy to T2D prevention at the population level.^146^ For example, analyses of changes in dairy intake over time have reported that increasing yogurt consumption is associated with lower subsequent T2D risk, whereas associations may differ by dairy subtype.^147^ The lactic acid bacteria involved in fermentation, including various *Lactobacillus* and *Bifidobacterium* species, can transiently colonize the gastrointestinal tract and positively interact with resident microbial communities as well as host immune pathways.^148^ Regular consumption of fermented dairy has been linked to a range of health benefits, including enhanced gut barrier function, and modulation of inflammatory responses, although effects likely depend on product formulation (e.g., added sugars), baseline metabolic status, and overall dietary pattern.^149^^,^^150^

Probiotics are defined as specific live microorganisms delivered via foods (including some fermented dairy products) or supplements in adequate amounts to confer a health benefit. In patients with T2D, recent systematic reviews and meta-analyses of randomized controlled trials indicate that probiotic supplementation can modestly improve glycemic control, including reductions in fasting glucose and HbA1c (with heterogeneity by strain composition, dose, duration, and baseline glycemic status).^151^ Food-matrix delivery may also be relevant: controlled trials of probiotic-containing fermented foods/drinks have reported improvements in diabetes-related outcomes in some settings, although durable long-term colonization is generally limited and benefits likely reflect ongoing intake and functional effects on microbial metabolism rather than permanent engraftment.^152^ Beyond transient microbial exposure, fermentation process itself can also generate bioactive compounds, such as peptides derived from milk proteins, which have demonstrated cardiometabolic benefits in experimental and clinical studies.^153^^,^^154^

### 4.6. Dietary patterns

Beyond individual nutrients and foods, dietary patterns exert holistic, synergistic effects on the gut microbiome and metabolic disease risk. The combined effects of the various components within a diet create a distinct microbial signature.^155^ The Mediterranean diet, characterized by a high intake of fruits, vegetables, legumes, whole grains, nuts, and olive oil, moderate consumption of fish and poultry, and low consumption of red meat and sweets, is a well-established paradigm for preventing metabolic diseases.^156^ Much of its benefits appear to be mediated through the gut microbiome: adherence to this dietary pattern is associated with increased microbial diversity and enrichment of SCFA-producing taxa such as *F. prausnitzii*, *Roseburia spp.*, *Bifidobacterium*, along with reductions in pro-inflammatory microbes, including certain *R. gnavus* strains.^157^^-^^159^ Its high fiber and polyphenol content provides abundant substrates for microbial fermentation, leading to the production of health-promoting metabolites.^32^ Notably, the protective association between Mediterranean diet adherence and reduced T2D and metabolic disease prevalence appears strongest in individuals with depleted abundance of *P. copri*, underscoring microbiome-dependent variation in dietary responses.^33^^,^^34^ Vegetarian and vegan dietary patterns similarly enrich beneficial fermenters while reducing production of harmful metabolites like TMAO.^160^ In contrast, the Western diet, characterized by a high intake of processed foods, refined carbohydrates, saturated fats, and sugar, and a paucity of fiber and polyphenols,^161^ drives microbial dysbiosis marked by reduced diversity, depletion of beneficial microbes, and expansion of pathobionts,^126^ as well as impaired gut barrier^162^ and subsequent translocation of pro-inflammatory bacterial components, such as LPS, triggering a state of chronic low-grade inflammation that underlies insulin resistance and metabolic disease.^26^

### 4.7. Microbiome-dependent variability and personalized nutrition

An important and rapidly growing area within diet–microbiome research concerns the striking inter-individual variability in metabolic responses to the same dietary pattern.^163^ This heterogeneity is increasingly understood to be shaped, in large part, by the unique taxonomic composition and functional capacity of each individual’s gut microbiome.^163^^,^^164^ These insights form the conceptual foundation for the emerging field of microbiome-informed personalized nutrition, in which dietary recommendations are tailored to an individual's unique biological profile, including their microbial profile, to optimize metabolic health.^164^^-^^166^ Evidence supporting this approach is mounting. For example, personalized models incorporating gut microbial data outperform conventional dietary guidelines in predicting postprandial glycemic responses across diverse populations, demonstrating the microbiome’s substantial predictive power for individual dietary effects.^167^ Likewise, large-scale analyses reveal that diet and the gut microbiome together explain more inter-individual variation in circulating metabolites than host genetics alone, underscoring the central role of microbial metabolism in shaping nutritional phenotypes.^168^ These findings suggest that, by characterizing an individual's microbial functional potential, it may become possible to anticipate metabolic responses to specific foods and design interventions with greater likelihood of success. For instance, in human dietary fiber interventions using barley kernel–based foods, improvements in glucose metabolism were observed predominantly in individuals with higher baseline *Prevotella* abundance (or higher *Prevotella-to-Bacteroides* ratio), whereas non-responders tended to have low baseline *Prevotella-to-Bacteroides* ratios—supporting the concept that baseline microbiome composition can stratify metabolic responsiveness to specific fiber types.^169^ More broadly, controlled prebiotic crossover studies demonstrate pronounced inter-individual variability in SCFA production in response to different prebiotics, with baseline microbial/metabolic state (e.g., baseline fecal SCFA levels and habitual fiber intake) helping explain who responds most strongly.^170^ Consistent with this, recent work in resistant starch supplementation shows that baseline gut microbial features and fiber intake can predict microbiota response, providing a tractable framework for matching fiber interventions to an individual’s microbiome profile.^171^ Despite this promise, substantial challenges remain. The gut microbiome is a highly dynamic ecosystem influenced by numerous factors beyond diet, including host genetics, medications, environmental exposures, stress, and physical activity. Robust microbial biomarkers, standardized analytical frameworks, and scalable intervention strategies will be essential for translating microbiome-based personalized nutrition into clinical and public health practice. Continued research is therefore critical to refine predictive models, establish causal mechanisms, and ensure the reliability and accessibility of microbiome-guided dietary recommendations.

## 5. Strain-level profiling and functional diversity

### 5.1. The importance of strain-level resolution

Most microbiome studies of T2D have examined microbial features at the species level or higher. However, species–level analyses implicitly assume functional uniformity within species, overlooking the substantial genomic and physiological heterogeneity that exists among strains.^111^ Individual strains within the same species can harbor distinct gene repertoires and exhibit divergent—or even opposing—effects on host physiology. Indeed, pathogenic mechanisms are often strain-specific: particular strains may be directly causal for disease outcomes, or the microbial functions relevant to host metabolic dysfunction may be carried out only by a subset of strains within a species. A classic illustration of this principle is *E. coli*, which comprises strains that range from benign (e.g., K-12) to highly pathogenic (e.g., enterohemorrhagic *E. coli* O157:H7) to probiotic (e.g., Nissle 1917).^172^^,^^173^ As a result, species-level summaries can obscure the specific microbial strains and functional pathways that drive disease-related phenotypes. These limitations underscore the need for strain-resolved profiling to uncover the true microbial contributors to T2D pathogenesis. To address these challenges, strain-resolved profiling methods infer within-species variation from shotgun metagenomic data using two complementary strategies: (i) marker/SNV-based approaches that track strain lineages via variation in species-specific markers, and (ii) pangenome/gene-content approaches that quantify strain-variable genes and pathways. These approaches are particularly important for T2D because clinically relevant signals can be strain-specific—for example, only subsets of strains may encode BCAA biosynthesis capacity, virulence-associated functions, or bile acid–modifying genes plausibly linked to insulin resistance and inflammation—thereby contributing to cross-cohort inconsistency in species-level associations.

### 5.2. Ecological and evolutionary drivers of strain diversity

Strain diversity reflects ecological adaptation to host environments and can be structured by geography, habitual diet, and inflammatory tone—factors that also vary systematically across T2D populations. Large-scale studies reveal that strains of common gut microbes often exhibit strong geographic structuring, reflecting local selective pressures—including long-term dietary patterns—that drive lineage-specific adaptation.^174^ For example, strain-level analyses of *E. rectale* and *P. copri* show distinct subclades associated with populations from North America versus China, whereas *F. prausnitzii* displays a more continuous gradient of variation that nonetheless correlates with host geography.^174^ These findings underscore that the “version” of a species carried by an individual may reflect adaptation to their habitual diet and lifestyle. Understanding strain-level variation is especially critical for T2D, a condition with strong dietary and inflammatory underpinnings. Both host diet^55^^,^^175^ and immune activity^176^ exert powerful selective pressures that shape within-species microbial diversity in the gut.^177^ By resolving the fine-scale diversity within microbial species, strain-level profiling offers a path toward identifying the true microbial drivers of metabolic dysfunction.

### 5.3. Strain-level signatures linked to type 2 diabetes

Recent work from our group underscores this point. Using Anpan, a suite of quantitative methods for high-resolution, strain-level profiling, we analyzed 8,117 shotgun metagenomes from a large international population.^38^ Anpan integrates three complementary population-level approaches to resolve within-species microbial diversity. First, linear models identify strain-specific genetic elements associated with host metabolic traits. Second, phylogenetic generalized linear mixed models delineate associations between subspecies lineages and host phenotypes while explicitly accounting for shared evolutionary history. Third, random-effects models estimate the likelihood that specific pathways are preferentially retained or lost among outcome-associated strains. Application of the phylogenetic generalized linear mixed models revealed that within-species phylogenetic diversity—rather than species-level abundance—explains inter-individual variation in T2D risk for 27 microbial species.^38^ For example, in *E. rectale*, a major butyrate producer, metabolic health was associated with the dominant subclade rather than overall species abundance, illustrating how species-level summaries can obscure clinically relevant heterogeneity. Similarly, random-effects modeling, which maps disease-associated functions to specific microbial lineages, showed that *P. copri* strains encoding BCAA biosynthesis pathways were enriched in individuals with T2D, whereas strains lacking this functional module were not—highlighting that strain-specific gene carriage, rather than species identity alone, drives metabolic disease associations. Finally, linear modeling of gene–strain relationships further demonstrated that clusters of *E. coli* strains carried genetic markers consistent with known pathogenic subtypes. Notably, several T2D-enriched clusters harbored gene families encoding virulence-associated functions—including adhesins, invasins, and toxins—suggesting that specific *E. coli* lineages with expanded pathogenic potential may contribute disproportionately to metabolic dysfunction.

### 5.4. Longitudinal strain tracking

While cross-sectional strain-resolved analyses have yielded important mechanistic insights, fully elucidating how host–microbiome interactions arise, evolve, and respond to perturbations requires longitudinal tracking, which introduces distinctive inferential, technical, and logistical challenges. Identifying identical strains across individuals can suggest person-to-person transmission, yet such patterns are not definitive evidence of direct contact because shared environmental reservoirs can produce parallel acquisition. For example, in wild baboons, demographic and environmental effects exerted stronger influences on strain sharing than social interactions, highlighting the difficulty of disentangling true transmission from shared exposure.^178^ Longitudinal designs are also critical for separating genetic influences from environmental confounding: in cross-sectional studies, genetic relatedness often covaries with cohabitation, diet, and lifestyle, obscuring heritability signals, whereas repeated sampling across natural changes in environment or behavior can reveal host genetic effects on microbial stability and responsiveness.^179^ Implementing such studies at scale remains challenging due to the high cost of serial shotgun metagenomics, the operational demands of frequent and well-controlled sampling, and the computational burden of analyzing large microbiome time-series. Additional complications arise in low-biomass environments, where samples are dominated by host DNA and require specialized laboratory methods and carefully tuned bioinformatic pipelines to achieve reliable strain-level resolution.^180^^,^^181^

## 6. Summary and future directions

Research at the intersection of the gut microbiome, diet, and metabolic disease has undergone a major transformation. Accumulating evidence supports that the gut microbiome is not only a correlate of metabolic health but can act as a mechanistic contributor linking environmental inputs, particularly diet, to host metabolic outcomes. Large-scale human studies consistently demonstrate a characteristic dysbiosis in T2D, marked by a depletion of beneficial, butyrate-producing bacteria and an overrepresentation of opportunistic pathogens. Mechanistic work has revealed that this altered ecosystem contributes to T2D pathophysiology through multiple intertwined pathways: dysregulated production of bioactive metabolites (e.g., SCFAs, BCAAs, and bile acids), compromised gut barrier integrity, and chronic, low-grade inflammation fueled by metabolic endotoxemia. Importantly, the field’s transition from species-level analyses to high-resolution, strain-resolved profiling has uncovered that many disease-associated functions are strain-specific, a layer of biological specificity that earlier studies could not capture. Beyond bacteria, emerging work on the archaeal, viral, and fungal components of the microbiome—including the virome and mycobiome—suggests additional pathways that may influence insulin resistance, glycemic control, and diabetes-related complications, but remain incompletely characterized.

A central priority is to move from cross-sectional case–control signatures to longitudinal, clinically anchored trajectories. Future cohorts should link multi-kingdom microbiome features to glycemic control over time (e.g., HbA1c trajectories, postprandial glycemia, insulin secretion or sensitivity indices), integrating high-resolution phenotyping such as continuous glucose monitoring and repeated sampling to capture dynamic microbial functions. Studies should also test whether microbiome features predict progression from normoglycemia to prediabetes and incident T2D, and whether they track *β*-cell failure versus predominantly insulin-resistant phenotypes (including within emerging T2D subtyping frameworks). A second priority is to define how microbiome alterations relate to diabetes complications and comorbidities—including diabetic kidney disease, NAFLD, cardiovascular outcomes, and chronic low-grade inflammation—and to distinguish microbial drivers from changes reflecting medication exposure, dietary change, or disease severity. A third priority is the development of microbiome-informed precision dietary approaches for prevention and intervention. This is particularly relevant given that dietary risk factors account for a substantial proportion of preventable T2D cases.^182^ Moreover, considerable interindividual variability exists in glycemic responses, particularly postprandial glucose excursions, and the gut microbiome may explain a meaningful fraction of this variability.^183^ Elucidating these relationships could establish a foundation for more personalized and effective dietary strategies for T2D prevention and management.

Addressing these aims will require (i) time-varying longitudinal models and causal inference frameworks that explicitly account for confounding by adiposity, diet, and medications (notably metformin and GLP-1–based therapies), and that can test mediation via microbial metabolites and host pathways; (ii) improved strain-resolved and function-first pipelines (e.g., pangenome/contig-level analysis and improved annotation of microbial “dark matter”) to identify actionable microbial targets; and (iii) T2D-relevant experimental platforms—including gnotobiotic models with defined consortia, intestinal organoids and gut–liver/gut–pancreas organ-on-chip systems, and microbiome–immune co-culture models—to validate barrier, endocrine (incretin), and inflammatory mechanisms. Finally, translational progress will depend on well-controlled intervention trials designed around T2D endpoints (continuous glucose monitoring-derived glycemic responses, insulin sensitivity, *β*-cell function, and complication biomarkers) while jointly measuring microbial activity (metatranscriptomics), metabolite flux (targeted/untargeted metabolomics), and multi-kingdom dynamics.

In parallel, existing human microbiome-targeted interventions provide important translational context. Human trials targeting the microbiome in T2D and related metabolic phenotypes have produced mixed and often modest results. Meta-analyses of randomized trials suggest that probiotics, prebiotics, and synbiotics can improve glycemic indices on average, but effect sizes are generally small and heterogeneous across formulations, doses, durations, and background diet/medication exposure.^184^ Fecal microbiota transplantation (FMT) provides proof-of-concept for causality in humans: lean-donor FMT delivered endoscopically has improved peripheral insulin sensitivity at ~6 weeks in metabolic syndrome, but benefits can be transient and not consistently sustained.^185^ Moreover, capsule-based FMT can achieve engraftment yet show neutral metabolic outcomes in some placebo-controlled trials, underscoring that engraftment does not guarantee clinical benefit with current protocols.^186^ Together, these findings motivate next-generation T2D intervention designs that incorporate microbiome-based stratification, multi-omics readouts, and clinically meaningful endpoints. Ultimately, the goal is to develop safe and effective microbiome-based strategies—precision nutrition, targeted prebiotics, next-generation probiotics/postbiotics, and microbiome-informed pharmacotherapy—supported by evidence on who benefits, through which microbial pathways, and in what clinical contexts.

## Data Availability

This manuscript does not involve data analysis.
